# Correlates of disability among primary care patients with common mental disorders and chronic medical conditions- a cross-sectional study from rural South India

**DOI:** 10.1007/s00127-024-02727-w

**Published:** 2024-07-23

**Authors:** Luke Joshua Salazar, Divya Hegde, Krishnamachari Srinivasan, Elsa Heylen, Maria L. Ekstrand

**Affiliations:** 1 Department of Psychiatry, St. John’s Medical College, Bengaluru, Karnataka 560034 India; 2 Division of Mental Health and Neurosciences, St. John’s Research Institute, Sarjapur Road, Bengaluru, Karnataka 560034 India; 3 Center for AIDS Prevention Studies, Division of Prevention Sciences, Department of Medicine, University of California, San Francisco, 94158 USA

**Keywords:** Depression, Anxiety, Noncommunicable diseases, Disability, Female

## Abstract

**Purpose:**

We examined the correlates of disability among people with non-communicable diseases (NCDs) and comorbid common mental disorders (CMDs) from rural India.

**Methods:**

The sample comprised 2,486 participants enrolled in a cluster randomized trial (cRCT), Healthier OPtions through Empowerment (HOPE). Participants were 30 years or older, with a diagnosis of major depressive disorder, dysthymia, generalized anxiety disorder, and/or panic disorder on the MINI-International Neuropsychiatric Interview, with hypertension, diabetes, dsylipidemia and/ or ischemic heart disease. Disability was measured with the 12-item version of WHODAS 2.0. The severity of depression and anxiety was measured using the Patient Health Questionnaire (PHQ-9) and the Generalized Anxiety Disorder Scale (GAD-7), respectively. Multiple linear regression analyses were used to examine associations.

**Results:**

The mean age was 59 ± 10.0 years, three quarters (1864) of the participants were female, and 64.0% were married. More than half of the participants had no formal education (57.9%). Most of the participants had two or more chronic medical conditions (73.0%). The mean disability score was 24.3. The mean depression score was 8.5, and the mean anxiety score was 6.7. Higher levels of disability were reported by participants ≥ 60 years of age, those with moderate and severe depression, and moderate anxiety. Among female participants, being unmarried was associated with greater disability. Male participants without formal education reported greater levels of disability.

**Conclusion:**

Higher severity of CMDs is significantly associated with higher levels of disability. For women, being unmarried and for men having no formal education was associated with higher levels of disability.

**Trial registration:**

ClinicalTrials.gov NCT02310932 [URL: https://clinicaltrials.gov/ct2/show/record/NCT02310932] registered on December 8, 2014, and Clinical Trials Registry India CTRI/2018/04/013001, registered on April 4, 2018. Retrospectively registered.

## Introduction

Non-communicable diseases (NCDs) and common mental disorders (CMDs)- depression and anxiety disorders- are among the leading causes of death and disability in India, and are increasing in prevalence. The Indian Council of Medical Research–India Diabetes (ICMR-INDIAB-17) study, a cross-sectional population-based survey which assessed a representative sample of individuals aged 20 years and older drawn from urban and rural areas, reported the overall weighted prevalence of diabetes mellitus (DM) at 11·4% (95% CI 10·2–12·5), hypertension (HT) at 35·5% (33·8–37·3), and dyslipidaemia (DLP) at 81·2% (77·9–84·5) [1]. The Global Burden of Disease (GBD) study estimated 23.8 million prevalent cases of ischemic heart disease (IHD) in India in 2016, 2.3 times the number from 1990, which contributed 17.8% of total deaths, and was the leading cause of disability adjusted life years (DALYs) at 8.7% [2]. Leading risk factors for DALYs attributable to IHD were high systolic blood pressure and high total cholesterol [2]. The GBD also estimated that the age-standardized DALY rate for DM in India increased by 39.6% from 1990 to 2016, which was the highest increase among major NCDs [3]. Prevalence of CMDs was also high according to the GBD, which estimated that in 2017 45·7 million people had depressive disorders and 44·9 million people had anxiety disorders [4]. The contribution of mental disorders to the total DALYs in India increased from 2·5% (2·0–3·1) in 1990 to 4·7% (3·7–5·6) in 2017, with depressive disorders contributing the most to the total mental disorders DALYs (33·8%, 29·5–38·5), followed by anxiety disorders (19·0%, 15·9–22·4) [4]. NCDs and CMDs are intimately related on multiple levels [5]. Epidemiological studies consistently demonstrate a strong association between the two groups of disorders with high rates of comorbidity [6–9]. Causal mechanisms implicated range from individual to societal, and operate bidirectionally between key risk factors, for both NCDs and CMDs [5, 10–15].

A few studies that have reported on disability in participants with NCDs and CMDs have shown a synergistic effect of the comorbidity of these health conditions on disability [16–19]. Kessler et al. [16], in a large nationally representative household survey from the United States reported on disability, mental disorders and four common chronic conditions: HT, arthritis, asthma, and ulcers. All four physical disorders were associated with significant role impairments in bivariate analyses. However, further analysis showed that these impairments were almost entirely confined to cases with comorbid mental disorders. Schmitz et al. [17], in a large nationally representative survey from Canada, reported that depression increased the odds ratio for functional disability from 2.1 in chronic conditions only to 6.3 in chronic conditions with comorbid depression, indicating a strong positive interaction. The World Mental Health (WMH) Survey Initiative, a large general population survey carried out in countries in all World Health Organization (WHO) regions, also reported that the odds of severe disability among those with both CMDs and chronic physical conditions were significantly greater than the sum of the odds of the single conditions [18]. An analysis of data from the United States National Health Interview Survey (NHIS), including a nationally representative sample of 30,022 adults, grouped participants into four disease categories- no DM and no major depression, major depression alone, DM alone, and DM and comorbid major depression [19]. With no DM and no major depression as reference and after adjusting for relevant covariates, the odds ratio of overall functional disability in individuals with DM and comorbid major depression (6.15) was significantly higher than the sum of the independent odds of functional disability in individuals with DM and major depression (5.48), which again, suggests a synergistic effect rather than an additive effect. Independent of disease category, the elderly (≥ 65 years), women, people with lower household income and those with less than high school education had significantly higher odds of functional disability.

In a community sample of participants with DM, Smith et al. found an increased likelihood of reporting functional disability for participants with high anxiety or depressive symptoms or both [20]. However, there was no synergistic interaction effect of anxiety and depression symptoms on level of functional disability. Depression has also been shown to predict future disability in patients with DM. In a longitudinal study which followed up 2733 participants with DM for a period of 5 years, for participants who were not depressed at baseline, the onset and persistence of depression predicted disability at 5 years [21].

To the best of our knowledge, there are no studies that examine the correlates of disability among people with NCDs and comorbid CMDs from low middle-income countries (LMICs), where the prevalence rates of both these health conditions are on the rise. There is also scarce research examining differences in the correlates of disability by gender. A study of these associations is relevant since differences across gender in the prevalence [22, 23], severity [24, 25], risk factors- including age and marital status [26–28], course [22, 23] and comorbidity [23] of CMDs are well documented in the literature. Gender-based differences also exist in the risk factors, prevalence, pattern and outcomes of NCDs [29–33]. This paper therefore aimed to examine the correlates of disability among people with CMDs and co-morbid NCDs from primary care in rural southern India.

## Methods

### Sample and study design

The data used in these analyses were from 2,486 participants enrolled in a cluster randomized clinical trial (cRCT), Healthier OPtions through Empowerment (HOPE) The objective of the trial was to examine the efficacy of the integrated management of mental health and chronic medical conditions using a collaborative care model in primary health centres (PHCs) in rural South India. The study was conducted in 49 PHCs in the rural Ramnagara district of Karnataka state in southern India. PHCs were randomly selected and were randomized 1:1 into the collaborative care and “enhanced” standard care arms. The collaborative care model consisted of two components: a clinic-based intervention and a community-based intervention, targeting the individual participants living in the catchment areas of the collaborative care model PHCs. As part of the clinic-based intervention, the participants received diagnostic testing and clinical treatment for both their mental illness and chronic disease by the PHC care team that included a physician, a nurse, and a pharmacist. All three were trained in comprehensive integrated mental health and physical health care by study-affiliated psychiatrists and community medicine physicians. During the intervention, the staff in collaborative care PHCs received support via weekly phone calls from consultant psychiatrists. The community-based intervention comprised of “healthy living” group meetings in an accessible community venue, designed to target risk factors important in the management of NCDs and CMDs. The first 12 sessions occurred weekly and were facilitated by a master’s level counsellor and co-facilitated by an Accredited Social Health Activist (ASHA). They were followed by nine monthly sessions in which ASHAs were encouraged to take the lead. ASHAs met with every participant’s family during bi-monthly home visits and encouraged them to support the participant’s new healthy lifestyle. The cRCT protocol has been described elsewhere [34].

Eligible participants were people living in the PHC catchment areas, 30 years or older, with a diagnosis of major depressive disorder, dysthymia, generalized anxiety disorder, and/or panic disorder on the MINI-International Neuropsychiatric Interview [35], co-diagnosed with HT, DM, and/or IHD, and able to consent to participate and be followed for 12 months. Prospective participants were evaluated for HT (elevated systolic blood pressure > 140 mmHg and/or diastolic blood pressure > 90 mmHg), DM (capillary blood sugar ≥ 160 mg/dl), and angina (positive response on the Rose Angina Questionnaire) [36] and/or being on treatment for HT, DM or IHD. At least one of these diagnoses was required to be eligible for enrolment. Participants unable to provide consent due to cognitive impairment (Modified Short Blessed Cognitive Scale > 7) [37], unable to provide contact information, and those on anti-depressants at the time of initial screening, were excluded from the study. The baseline data used here were collected between May 2015 and November 2018.

### Measures

All measures were collected via face-to-face interviews conducted by trained interviewers.

#### Disability

Disability was measured with the 12-item version of WHO Disability Assessment Schedule 2.0 (WHODAS 2.0), a brief, widely used assessment of overall functioning [38]. Participants mark on a 5-point scale ranging from 1 ‘None’ to 5 ‘Extreme – cannot do’ how much difficulty they had in the past 30 days with various physical (e.g. standing for > 30 min.), cognitive (e.g. learning new task) or social tasks (e.g. maintaining a friendship). There are 6 different functioning domains - cognition, mobility, self-care, getting along, life activities (household and work/school) and participation. Answers were summed over all items, as per the ‘simple scoring’ instructions in the WHODAS manual resulting in a range from 12 to 60 [38]. The WHODAS 2.0 is grounded in the conceptual framework of the International Classification of Functioning, Disability and Health (ICF). The ICF defines disability as “a decrement in each functioning domain” and conceptualizes disability from the perspective of the biopsychosocial model. It therefore recognizes that disability is multidimensional and is the product of an interaction between attributes of an individual and features of the person’s physical, social and attitudinal environment. In psychometric analysis the WHODAS 2.0 structure has been shown have high internal consistency [38]. In the current sample, internal consistency was α = 0.89.

#### Severity of depressive and anxiety symptoms

The nine-item version Patient Health Questionnaire (PHQ-9) was used to assess severity of depression in the past 2 weeks [39]. In our sample the internal consistency was α = 0.81. Responses are measured on a 0 (not at all) to 3 (nearly every day) scale and are summed over all items. Participants were classified into minimal (0–4), mild (5–9), moderate (10–14), and severe (15–27) depression categories.

The Generalized Anxiety Disorder Scale (GAD-7) was used to assess severity of anxiety in the past 2 weeks [40]. In our sample the internal consistency was α = 0.84. Responses were measured on a 0 (not at all) to 3 (nearly every day) scale and summed over all seven items. Participants were classified into minimal (0–4), mild (5–9), moderate (10–14), and severe (15–21) anxiety categories.

#### Hypertension

Systolic and diastolic blood pressure (BP) was measured using a standardized protocol. Two measurements were taken. HT was defined as showing elevated systolic BP (SBP) ≥ 140 mmHg and/or diastolic BP (DBP) ≥ 90 mmHg on both measurements or self-report confirmed by showing a prescription or medications [41].

#### Diabetes Mellitus

HbA1c was selected for the measurement as it is not affected by short term dietary changes. DM was defined as HbA1c ≥ 6.5 and/or self-report confirmed by a prescription or medications [42].

#### Dyslipidemia

Lipids (total cholesterol, Low Density Lipoprotein (LDL), High Density Lipoprotein (HDL) and triglycerides) were measured. DLP was defined as: LDL > 190 mg/dl without DM and > 70 mg/dl with DM and /or self-report confirmed by showing a prescription or medications [43].

#### Ischemic heart disease

During screening, cardiac risk was assessed using Rose Angina questionnaire (RAQ) [36]. It has two parts- Part A and Part B. A positive response to either Part A or Part B was considered positive for IHD. A positive RAQ result had to be confirmed via ECG for a participant to have a diagnosis of IHD.

#### Chronic Medical conditions

The sum of the above dichotomous variables for HT, DM, DLP and IHD was taken to create a 1–4 variable representing the number of chronic medical conditions. As per the inclusion criteria, participants had to have at least one chronic medical condition.

#### Demographics

Sociodemographic variables assessed included age, gender, marital status (not married included never married, divorced/separated and widowed individuals), education status, and income.

### Ethical considerations

Ethics approval was obtained from the Institutional Ethical Review Board at St. John’s Medical College, Bangalore and Committee on Human Research, University of California, San Francisco. Eligible participants received information about the study verbally as well as in written form including information about the intervention, study protocol, randomization process, time commitment and potential risks and benefits and provided written consent. Illiterate participants had the option of providing either verbal consent or a thumbprint. In such cases, a witness, unaffiliated with the study, also signed the consent form.

### Analyses

Continuous variables were described via the mean with standard deviation, and categorical variables as frequencies and percentages. Differences between men and women on demographic, categorical variables were tested via χ^2^-test. Comparison of disability, depression and anxiety score by demographic and clinical variables were performed using independent t-test and Analysis of Variance (ANOVA). Unadjusted and adjusted regression coefficients with 95% confidence interval were calculated using univariate and multivariate analysis, respectively. Variables that were significant in the univariate analysis, were included in the multivariate analysis in a single step. P value less than 0.05 was considered statistically significant. Preliminary analysis was carried out in the entire study sample. Results showed a significant interaction of gender by marital status and gender by education. Based on our findings and significant gender differences in relation to severity of depression noted in the literature [24, 25], a further analysis was carried out stratified by gender. All analyses were carried out using SPSS version 25.0.

## Results

A total of 2,678 people were eligible for the study. However, 192 did not consent, yielding a final baseline sample of 2,486. The mean age was 59 ± 10.0 years, three quarters of the participants were female, and 64.0% were married. Baseline characteristics of the participants, by gender, are presented in Table 1.

**Table 1:** Baseline characteristics of participants, by gender

	Overall (*n* = 2486)		Male (*n* = 622)		Female (*n* = 1864)		χ^2^/t and *p*-value
	*n*	%	*n*	%	*n*	%	
Age > 60	1070	43.0	365	58.7	705	37.8	χ^2^ = 82.8; *p* < 0.001
Married	1591	64.0	585	94.1	1006	54.0	χ^2^ = 325.2; *p* < 0.001
No formal education	1438	57.9	181	29.1	1257	67.4	χ^2^ = 281.1; *p* < 0.001
HH income ≤ 5000 IRS	1770	71.2	387	62.2	1383	74.2	χ^2^ = 32.6; *p* < 0.001
HT (*n* = 2482)	1796	72.4	424	68.4	1372	73.7	χ^2^ = 6.5; *p* = 0.011
DLP (*n* = 2472)	1634	66.1	417	67.1	1217	65.7	χ^2^ = 0.4; *p* = 0.523
DM (*n* = 2476)	1699	68.6	449	72.4	1250	67.3	χ^2^ = 5.5; *p* = 0.018
IHD (*n* = 2459)	318	12.9	89	14.5	229	12.4	χ^2^ = 1.7; *p* = 0.189
Number of comorbid conditions							χ^2^ = 5.0; *p* = 0.173
1	670	27.0	156	25.1	514	27.6	
2	784	31.5	210	33.8	574	30.8	
3	919	37.0	221	35.5	698	37.4	
4	113	4.5	35	5.6	78	4.2	
Depression							χ^2^ = 6.7; *p* = 0.035
Minimal/mild	1629	65.6	433	69.6	1196	64.2	
Moderate	627	25.2	134	21.5	493	26.4	
Severe	230	9.3	55	8.8	175	9.4	
Anxiety							χ^2^ = 6.4; *p* = 0.040
Minimal/mild	1957	78.7	509	81.8	1448	77.7	
Moderate	439	17.7	89	14.3	350	18.8	
Severe	90	3.6	24	3.9	66	3.5	
Disability score: mean (SD) (12–60)	24.3	(7.7)	23.6	(8.2)	24.5	(7.5)	t=-2.59; *p* = 0.010
Depression score: mean (SD) (0–27)	8.5	(4.1)	8.1	(4.3)	8.7	(4.1)	t=-3.18; *p* = 0.001
Anxiety score: mean (SD) (0–21)	6.7	(3.7)	6.4	(3.8)	6.9	(3.7)	t=-3.03; *p* = 0.002

HH, Household; HT, Hypertension; DLP, Dyslipidemia; DM, Diabetes Mellitus; IHD, Ischemic Heart Disease; SD, Standard Deviation

More than half of the participants had no formal education (57.9%). A large majority of participants had DM (68.6%) or HT (72.4%) while 12.9% had IHD. Majority of the participants had two or more chronic medical conditions (73.0%). More than half (65.6%) of the participants had minimal to mild depression and 25.2% had moderate depression. More than three quarters (78.7%) of the participants had minimal to mild anxiety. The mean disability score as measured by WHODAS 2.0 was 24.3 (SD = 7.7). The mean depression score as measured by PHQ-9 was 8.5 (SD = 4.1), and the mean anxiety score measured by GAD-7 was 6.7 (SD = 3.7).

**Table 2:** Disability scores by demographic and clinical variables, by gender

Variables	Male (*n* = 622) Mean ± SD	Female (*n* = 1864) Mean ± SD
Age		
<=60	22.6 ± 7.8^*^	24.1 ± 7.6^***^
> 60	24.3 ± 8.4	25.3 ± 7.3
Marital status		
Not married	22.3 ± 8.6	25.1 ± 7.7^**^
Married	23.7 ± 8.2	24.1 ± 7.4
Income		
<=5000	23.7 ± 8.3	24.4 ± 7.5
> 5000	23.3 ± 8.0	24.8 ± 7.8
Education		
No formal education	25.5 ± 8.8^***^	25.0 ± 7.6^***^
Formal education	22.8 ± 7.8	23.6 ± 7.3
Number of comorbid conditions		
1	23.1 ± 7.6	24.6 ± 7.5
2	23.7 ± 8.7	24.1 ± 7.4
3	23.5 ± 8.3	24.7 ± 7.6
4	25.1 ± 7.4	25.9 + 8.3
HT		
Absent	24.1 ± 9.2	23.8 ± 7.1^*^
Present	23.3 ± 7.6	24.8 ± 7.7
DM		
Absent	23.3 ± 7.7	24.7 ± 7.7
Present	23.7 ± 8.4	24.4 ± 7.5
DLP		
Absent	23.2 ± 7.6	24.8 ± 7.7
Present	23.7 ± 8.5	24.4 ± 7.5
IHD		
Absent	23.2 ± 8.0^**^	24.3 ± 7.4^**^
Present	25.7 ± 8.8	25.9 ± 8.0
Depression score		
Minimal / mild	21.8 ± 7.3^a***^	22.5 ± 6.6^a***^
Moderate	26.5 ± 8.8^b^	26.8 ± 7.1^b^
Severe	30.3 ± 7.9^c^	32.3 ± 8.0^c^
Anxiety score		
Minimal / Mild	22.5 ± 7.8^a***^	23.2 ± 6.9^a***^
Moderate	28.1 ± 8.0^b^	27.9 ± 7.2^b^
Severe	30.0 ± 9.2^b^	35.8 ± 8.7^c^

HT, Hypertension; DLP, Dyslipidemia; DM, Diabetes Mellitus; IHD, Ischemic Heart Disease

Comparison of disability scores using independent t test and ANOVA within males and females separately

^*^p < 0.05, ^**^p < 0.01, ^***^p < 0.001 (for overall test of between groups effect for ANOVA)

^a, b, c^ groups with different superscript differ significantly from each other, using Bonferroni correction

**Table 3:** Associations of demographic and health-related variables with WHODAS 2.0 disability score in males and females, multiple linear regression results

	Male (n = 615)		Female (n = 1843)	
	Adjusted b, 95% C.I.	*p*-value	Adjusted b, 95% C.I.	*p*-value
Age > 60	1.65 (0.42; 2.88)	0.009	1.15 (0.49; 1.81)	0.001
Married	-		-0.74 (-1.38; -0.10)	0.022
Educated	-2.00 (-3.33; 0.66)	0.003	-0.58 (-1.24; 0.09)	0.090
HT	-		0.38 (-0.33; 1.08)	0.293
IHD	1.67 (-0.04; 3.38)	0.056	0.90 (-0.03; 1.83)	0.059
Depression score				
Minimal/Mild	Ref		Ref	
Moderate	3.35 (1.88; 5.18)	< 0.001	3.38 (2.59; 4.18)	< 0.001
Severe	6.30 (3.49; 9.11)	< 0.001	6.68 (5.35; 8.02)	< 0.001
Anxiety score				
Minimal/Mild	Ref		Ref	
Moderate	2.19 (0.10; 4.27)	0.040	1.84 (0.90; 2.78)	< 0.001
Severe	2.89 (-0.84; 6.63)	0.129	7.85 (5.91; 9.78)	< 0.001

^b^, linear regression coefficient; CI, confidence interval; HT, Hypertension; IHD, Ischemic Heart Disease

Bivariate analyses of disability scores by demographic and clinical variables are presented in Table 2. Among males and females, the mean disability score was significantly higher in participants > 60 years of age, participants who did not receive formal education, with IHD and participants who had moderate to severe depression or anxiety (all *p* < 0.05). In addition, among females it was noted that disability score was significantly higher in those with HT and unmarried participants.

Results from multiple linear regression analyses that included all significant variables from Table 2 as covariates are presented in Table 3, stratified by gender. In these adjusted analyses the bivariately observed associations for males regarding age, education, moderate and severe depression and moderate anxiety remained significant, but IHD and severe anxiety were not significant. Among female participants, higher age, unmarried status, moderate and severe depression and anxiety were still significantly associated with greater disability. But education, HT and IHD were no longer significant in the multivariate model.

## Discussion

Higher levels of disability were reported by the elderly and those with moderate and severe depression, as well as those with moderate anxiety. In addition, among female participants, being unmarried was associated with greater disability. Male participants without formal education reported greater levels of disability.

Our finding of higher disability among older participants is in agreement with several earlier studies including from India [44–49]. Age has also been found to moderate the relationship between number of chronic diseases and physical functioning, with older participants having more physical functioning difficulties with increasing number of diseases [45, 46]. Additionally, Chou et al. found that for the NCDs included in our study, higher increments of disability occur in later stages of the disease [50], probably due to the longer duration of disease which leads to an accruement of complications. This may explain why there was no significant association between the number of NCDs and disability among our participants.

In the present study, being unmarried was associated with an increased disability score in women but not among men. This is consistent with previous epidemiological studies from rural India which demonstrate that, for both men and women as a group, being unmarried or widowed was associated with higher functional disability [46, 47]. A multivariate decomposition analysis of data from the first wave of the Longitudinal Ageing Study in India showed that marital status contributed 13% and 10% to the gender gap (women > men) in difficulty in activities of daily living and instrumental activities of daily living, respectively [51]. Marital status has also been found to moderate the influence of depressive symptoms and chronic diseases on functional limitations, for both men and women as a group [52]. In rural India, it is likely that social norms that determine gender roles and disempowerment of women [53], may have a bearing on disability. Studies from India demonstrate that nationwide, single women have low levels of empowerment, including autonomy in taking decisions regarding healthcare and accessing healthcare facilities [54], which could lead to higher levels of disability. This is especially true for widowed women [55, 56], who constituted most of the unmarried women in our sample. Additionally, our sample contained very few unmarried men (6% of men vs. 46% of women), which could at least partially explain the gender difference we found because the study may not have been sufficiently powered to detect a similar difference in men.

Having no formal education was significantly associated with disability only in male participants. This is consistent with previous research which has shown low education levels to be associated with higher disability in samples from India and abroad among both men and women [46, 51, 57, 58]. It is likely that this association between educational attainment and disability is driven by several personal and contextual factors- lower-educated men may have fewer job options, less health literacy, difficulty in effectively utilizing their social and economic resources to manage chronic diseases, resulting in higher disability.

We found that participants with moderate and severe depression and/or anxiety had significantly higher odds of disability than those reporting minimal or mild symptoms of these CMDs, which increased with increasing severity of these CMDs. The results from our study underscore the importance of the severity of depression and anxiety as correlates of disability among participants with NCDs, which, to the best of our knowledge, has not previously been reported. Disability is a multidimensional construct as shown by the results reported here. The 12-item version of WHODAS 2.0 contains six latent constructs: cognition, mobility, self-care, getting along, life activities, and participation. Studies have also demonstrated that this version of WHODAS 2.0 can represent different combinations of several contributing factors [59]. Thus, it is likely that, among our participants, NCDs and CMDs contributed differentially to the domains of disability captured by this measure, resulting in higher disability scores among participants reporting higher severity of anxiety and depression. Additionally, CMDs may interact with NCDs through complex physiological, psychological, behavioural and environmental mechanisms operating at multiple levels to cause disability [5]. For example, depression impairs cognition and motivation which in turn can interfere with vital self-care processes in diabetics, like medication adherence. This can lead to DM-related complications such as diabetic foot, which can contribute to disability. Randomized controlled trials that have examined the efficacy of interventions for CMDs among participants in primary care settings have shown positive results for reduction of disability [60–62]. However, randomized controlled trials that have examined the efficacy of interventions for CMDs and comorbid NCDs among participants in primary care have not demonstrated the same benefit [63, 64]. Recent studies from India that have tested the efficacy of collaborative care interventions in participants with DM and CMDs have demonstrated positive results in terms of modifying these chronic diseases that are associated with disability [65, 66].

Future research would benefit from a focus on the explication of biological and behavioral mechanisms and intermediate factors that mediate sociodemographic predictors and CMDs with disability, in patients with NCDs. It might also be useful to examine the effect of treating both comorbid CMDs and NCDs on disability within the framework of collaborative care models.

### Strengths and limitations

The present study had multiple strengths, including a large community-based sample from rural India. Furthermore, in most studies on disability among participants diagnosed with NCDs and CMDs, the diagnosis of an NCD was based on the participant’s self-report [16–20], whereas for most of the participants in the present study, the diagnosis of an NCD was based on laboratory investigations. Geographical limitations preclude us from generalizing these findings to other regions. All the participants had CMD and NCD, therefore our findings cannot be generalized to people with only CMD or only NCD or neither condition. Finally, since the data in these analyses were cross-sectional, cause-effect relationships could not be established and need to be examined in future studies.

## Conclusion

In this rural population of people with CMD and co-morbid NCD conditions, reporting moderate to severe depression and moderate anxiety levels were significantly associated with higher levels of disability. In addition, being unmarried was associated with higher levels of disability among female participants. This information provides important targets to clinicians and researchers for future interventions to reduce the burden of disability in rural India.
